# Embryos are largely understudied in a representative sample of journals in conservation physiology

**DOI:** 10.1093/conphys/coag006

**Published:** 2026-02-18

**Authors:** Patrice Pottier, Nicholas C Wu, Madison L Earhart, Malgorzata Lagisz, Katharina Alter, Rafael Angelakopoulos, Avishikta Chakraborty, Zara-Louise Cowan, Shaun S Killen, Jamie C S McCoy, Estefany Caroline Guevara-Molina, Marta Moyano, Amanda K Pettersen, Luca Pettinau, Daniel M Ripley, Bao-Jun Sun, Ramakrishnan Vasudeva, Katharina Ruthsatz

**Affiliations:** Department of Biological and Environmental Sciences, Faculty of Science, University of Gothenburg, Medicinaregatan 7B, 41390, Gothenburg, Sweden; Evolution & Ecology Research Centre, School of Biological, Earth and Environmental Sciences, University of New South Wales, Kensington campus, Sydney, NSW 2052, Australia; Division of Ecology and Evolution, Research School of Biology, The Australian National University, 46 Sullivans Creek Rd, Acton, ACT 2601, Australia; Centre for Terrestrial Ecosystem Science and Sustainability, Harry Butler Institute, Murdoch University, 90 South Street, Murdoch, WA 6150, Australia; School of Environmental and Conservation Science, Murdoch University, 90 South Street, Murdoch, WA 6150, Australia; Department of Zoology, University of British Columbia, 6270 University Blvd, Vancouver, BC V6T 1Z4, Canada; Evolution & Ecology Research Centre, School of Biological, Earth and Environmental Sciences, University of New South Wales, Kensington campus, Sydney, NSW 2052, Australia; Department of Biological Sciences, Faculty of Science, The University of Alberta, Edmonton, AB, Canada; Department of Marine Biology, Institute of Biological Sciences, University of Rostock, Albert Einstein Straße 3, 18057 Rostock, Germany; Laboratory of Genetics, Comparative and Evolutionary Biology, Department of Biochemistry and Biotechnology, University of Thessaly, Campus Viopolis, 41500 Larissa, Greece; Division of Biosciences, Department of Genetics, Evolution and Environment, Centre for Life’s Origins and Evolution, University College London, Gower Street, London WC1E6BT, UK; Department of Biological and Environmental Sciences, University of Gothenburg, Kristineberg Marine Research Station, Kristineberg 566, Fiskebäckskil 451 78, Sweden; School of Biodiversity, One Health and Veterinary Medicine, University of Glasgow, Glasgow G12 8QQ, United Kingdom; School of Biosciences and Bateson Centre for Disease Mechanisms, University of Sheffield, Sheffield S10 2TN, United Kingdom; Laboratory of Behavior and Evolutionary Physiology, Department of Physiology, Institute of Biosciences, University of São Paulo, Rua do Matão, travessa 14, no. 101, Cidade Universitária – Butantã, São Paulo, SP, 05508-900, Brazil; Norwegian Institute for Water Research (NIVA), Økernveien 94, 0579 Oslo, Norway; Center for Coastal Research, University of Agder, Universitetsveien 25, 4604 Kristiansand, Norway; School of Life and Environmental Sciences, The University of Sydney, Heydon Laurence Building, Science Road, Camperdown, Sydney, NSW 2006, Australia; Institute for Marine and Antarctic Studies, University of Tasmania, 20 Castray Esplanade, Battery Point, Hobart, TAS 7004, Australia; Department of Animal Health and Welfare, Wageningen Livestock Research, Wageningen University and Research, De Elst 1, 6708 WD, Wageningen, Netherlands; Marine Biology Laboratory, New York University Abu Dhabi, Saadiyat Island, Abu Dhabi, United Arab Emirates, PO Box 129188; Key Laboratory of Animal Biodiversity Conservation and Integrated Pest Management, Institute of Zoology, Chinese Academy of Sciences, 100101 Beijing, China; Faculty of Environment, Science and Economy, Centre for Ecology and Conservation, University of Exeter, Penryn Campus, Penryn TR10 9FE, United Kingdom; Zoological Institute, Technische Universität Braunschweig, Mendelssohnstraße 4, 38106 Braunschweig, Germany; Ecology, Evolution and Development Group, Department of Wetland Ecology, Doñana Biological Station (CSIC), Calle Américo Vespucio 26, 41092 Sevilla, Spain

**Keywords:** Early-life stages, egg, embryonic development, environmental change, environmental stress, global change, life history, literature review, reproduction, systematic review map

## Abstract

Understanding how animals respond to environmental stressors across their life cycle is essential for predicting species' vulnerability to climate change. Here, we systematically reviewed the literature to quantify the variation in research effort on different life stages in the field of conservation physiology. Specifically, we reviewed experimental studies measuring physiological and life-history responses to climatic stressors across three representative scientific journals: *Conservation Physiology*, *Journal of Thermal Biology* and *Journal of Experimental Biology*. Our systematic map of 1276 studies revealed a pronounced underrepresentation of studies on embryos, representing only 8% to 9% of studies. This pattern was remarkably consistent across all axes considered (i.e. journals, taxonomic groups, physiological traits and environmental stressors). We also found that 80% of studies only investigated single life stages, and over 5% of studies did not clearly report the life stage(s) used. Despite the increasing recognition of the ecological importance and sensitivity of early life stages to environmental stressors, we found no evidence that research on embryos has gained traction over the past decade (2013–2024). We argue that these ontogenetic biases likely reflect a combination of historical precedents and enduring methodological and logistical constraints that continue to shape research agendas. To build a more holistic understanding across the life cycle, we: (i) call for a paradigm shift placing embryos at the center of experimental agendas, (ii) outline emerging methodological advances that increase the feasibility of research on early life stages, (iii) demonstrate how studies on embryos align with ethical considerations for animal research, (iv) highlight perspectives for future evidence syntheses and study reporting and (v) promote investigations of the mechanisms underlying physiological variation across ontogeny. Closing the ontogenetic gap will be key to improving our ability to predict population-level impacts of climate change and guiding more effective conservation and management interventions.

## Introduction

How animals respond to environmental change is a central question in ecophysiology, particularly in the face of ongoing climate change. By investigating the physiological mechanisms underlying organismal responses to climatic stressors, conservation physiology provides essential insights for predicting species resilience and guiding conservation strategies (Wikelski and Cooke, 2006; Cooke and O’Connor, 2010; Cooke *et al*., 2013). However, a key aspect that often remains overlooked is the need to understand these responses across all life stages of a species (Radchuk *et al*., 2013; Kingsolver and Buckley, 2020). A growing body of evidence suggests that the effects of climate change, such as rising temperatures and increased thermal variability, can vary significantly across life stages (e.g. Kingsolver *et al.*, 2011; Levy *et al.*, 2015; Truebano *et al.*, 2018; Dahlke *et al.*, 2020; Kingsolver and Buckley, 2020; Sales *et al.*, 2021; Ma *et al.*, 2024). Differences in sensitivity to environmental stressors across life stages can have profound implications for population persistence and species survival, making it essential to understand the stage-specific effects of climatic stressors, as they collectively shape population demography, dynamics and long-term viability under climate change (Radchuk *et al.*, 2013; Levy *et al.*, 2015; Kingsolver and Buckley, 2020).

Early life stages, such as embryos and larvae, are often hypothesized to be the most vulnerable due to their limited capacity for thermoregulation and heightened sensitivity to environmental fluctuations (Byrne, 2012; Huey *et al.*, 2012; Pandori and Sorte, 2019; Bodensteiner *et al.*, 2021; Burggren, 2021; Cheng *et al.*, 2023). These assumptions are supported by empirical data encompassing both ecto- and endothermic taxa (e.g. mammals; Zhao *et al.*, 2020); birds (Mainwaring *et al.*, 2017); amphibians (Ruthsatz *et al.*, 2022); reptiles (Sun *et al.*, 2021); fishes (Dahlke *et al.*, 2020); insects (Bowler and Terblanche, 2008); and aquatic invertebrates (Pandori and Sorte, 2019; but see Hamdoun and Epel, 2007; De Bonville *et al.*, 2025). However, recent comparative analyses have provided evidence that early life stages may be understudied relative to adults in conservation physiology, potentially overlooking key windows of climate vulnerability (Weaving *et al.*, 2022; Pottier *et al.*, 2022b; Ruthsatz *et al.*, 2024). For example, Pottier *et al.* (2022b) concluded that very little research has been conducted on the plasticity of thermal tolerance in ectotherm embryos, while Weaving *et al.* (2022) found that data on adults were more abundant than those on early life stages in insects. In contrast, a meta-analysis by Pandori and Sorte (2019) found research on early life stages (embryos and larvae) of aquatic invertebrates were more prevalent than research conducted on adults in the context of climate warming. However, the extent to which these ontogenetic biases are generalizable remains unclear, as there is currently no comprehensive evidence on how research effort varies across different life stages in conservation physiology. To address this gap, we systematically assessed for potential biases in research effort on different life stages across three conservation physiology journals.

In this study, we aimed to quantify the research effort dedicated to different life stages in the experimental conservation physiology literature. As a case in point, we conducted a systematic review map of studies published in three key journals that are likely representative of the literature published in the field: *Conservation Physiology*, *Journal of Thermal Biology* and *Journal of Experimental Biology*. We focused on animal physiology and life-history responses to climatic stressors and categorized studies by life stages, taxa and climatic stressors to assess the heterogeneity of patterns in published research. We sought to identify gaps in the representation of specific life stages, predicting that research on embryos would be underrepresented compared to studies on larvae/juveniles and adults. By highlighting this potential imbalance, we aimed to encourage research that is more inclusive of all stages in conservation physiology. We conclude by providing recommendations and perspectives on integrating underrepresented life stages, aiming to expand our understanding of population sensitivity to global change—knowledge essential for informing more effective conservation and management strategies.

## Materials and Methods

### Registration and reporting

This study was pre-registered prior to data extraction (Pottier *et al.*, 2024b). While we mostly followed our plans, we acknowledge a few minor deviations (see ‘Deviations from registration’). We followed MeRIT (Nakagawa *et al.*, 2023), CRediT (McNutt *et al.*, 2018) and Dragon Kill Points (Martinig *et al.*, 2026) guidelines to report authorship contributions (Tables S2 and S3). We also followed recommendations for reporting the title, abstract and keywords of this study and maximize indexing in search engines and databases (Pottier *et al.*, 2024a). All data, code and materials are freely available at https://github.com/p-pottier/Cons_phys_life_stages and permanently archived in Zenodo (Pottier *et al.*, 2026).

### Systematic review

We aimed to obtain a representative sample of experimental studies in animal conservation physiology. Specifically, we focused on studies assessing animal responses to climate change.

We systematically reviewed the literature to find studies that experimentally manipulated climatic stressors and measured physiological and/or life-history traits of non-human animals. Therefore, we excluded reviews, meta-analyses, field observations, theoretical studies, editorials and other study types without experimental manipulations. Note, however, that we included experimental studies performed in both laboratory and field settings, as long as climatic stressors were experimentally manipulated (e.g. altering operative body temperatures by adjusting nest depth of turtle eggs in the field). We focused on whole-organism non-human animals, hence excluded humans, plants, microorganisms or isolated cells and organs. Relevant climatic stressors included temperature, oxygen/carbon dioxide (O_2/_CO_2_), pH, salinity and humidity/water availability. We also included studies manipulating other stressors with climatic relevance, such as diet restriction and UV radiation levels. We only focused on physiological or life-history traits (environmental tolerance or preference, energetics and metabolism, development, immunity and stress physiology, osmoregulation, reproduction and cardiovascular physiology), to gain an overview of the field of conservation physiology broadly defined. Therefore, we excluded studies focusing on behavioural traits, morphology, ecological interactions or biodiversity measures, for instance.

PP performed the literature searches on 16 October 2024 in Web of Science (core collection, UNSW subscription). We did not use other databases because our searches were targeted to specific journals, so there is little discrepancy in results between databases. Searches were designed to capture studies manipulating climatic stressors in the journals *Conservation Physiology*, the *Journal of Thermal Biology* and the *Journal of Experimental Biology*.

Due to the large number of studies published across all journals in the past ten years, we could not expand the scope of the literature review to additional journals without altering the scope of the study (e.g. restricting the range of taxa or physiological traits). However, we were particularly interested in assessing how research effort on different life stages varied across taxa, traits and environmental stressors (Pottier *et al.*, 2024b). Therefore, our searches were not intended to be comprehensive, and these three journals were used as a case in point to assess the validity of informal statements made about the under-prevalence of experimental research on embryonic stages. The sample of studies we obtained is intended to provide a sample that is likely representative of the broader literature in the field of climate-related conservation physiology, yet carries limitations (see Discussion).

Searches were designed as follows:

*Conservation Physiology* (600 results): IS = (“2051–1434”) AND TS = (“climat*” OR “global change” OR “global warming” OR “environmental change” OR “temperature*” OR “therm*” OR “hypotherm*” OR “hypertherm*” OR “warm*” OR “heat*” OR “cold*” OR “cool*” OR “hot” OR “solar” OR “UV” OR “oxygen*” OR “hypoxi*” OR “hyperoxi*” OR “normoxi*” OR “anox*” OR “CO2” OR “carbon dioxide” OR “hypercapn*” OR “pH” OR “acidifi*” OR “salinity” OR “salt*” OR “humid*” OR “water” OR “drought*” OR “dry*” OR “dessicat*” OR “dehydr*” OR “rainfall” OR “moisture” OR “arid*”).

*Journal of Thermal Biology* (2136 results): IS = (“0306–4565”) AND PY = (2013–2024).

*Journal of Experimental Biology* (2145 results): IS = (“1477–9145”) AND TS = (“climat*” OR “global change” OR “global warming” OR “environmental change” OR “temperature*” OR “therm*” OR “hypotherm*” OR “hypertherm*” OR “warm*” OR “heat*” OR “cold*” OR “cool*” OR “hot” OR “solar” OR “UV” OR “oxygen*” OR “hypoxi*” OR “hyperoxi*” OR “normoxi*” OR “anox*” OR “CO2” OR “carbon dioxide” OR “hypercapn*” OR “pH” OR “acidifi*” OR “salinity” OR “salt*” OR “humid*” OR “water” OR “drought*” OR “dry*” OR “dessicat*” OR “dehydr*” OR “rainfall” OR “moisture” OR “arid*”) AND PY = (2013–2024).

Note that a more general search (i.e. no key terms on climatic stressors) was used for the *Journal of Thermal Biology* because all studies in this journal are focused on temperature, one of the key climatic stressors of interest. We restricted our searches to articles published after 2013 (when the journal *Conservation Physiology* was founded) to maintain a comparable timespan between journals. All searches were also filtered to article document types, to exclude reviews, notes and editorials.

We identified 4881 bibliographic records from these journals. All bibliographic records were combined and deduplicated in R (v. 4.4.2) (R Core Team (2025)) by PP using the litsearchr (v. 1.0.0) (Grames *et al.*, 2019) and synthesisr (v. 0.3.0) (Westgate and Grames, 2020) packages. A total of 4868 unique bibliographic records were identified.

K.A., R.A., A.C., Z.-L.C., M.L.E., S.S.K., J.C.S.M., E.C.G.-M., M.M., A.K.P., L.P., D.M.R., B.-J.S., R.V. and N.C.W. independently screened studies for eligibility based on their titles, abstracts and keywords in Rayyan QCRI (Ouzzani *et al.*, 2016) using the decision trees in Fig. S1. When eligibility criteria could not be assessed solely based on the title, abstract or keywords, we inspected the full article. In total, we identified 1276 relevant studies.

### Data extraction

K.A., R.A., A.C., Z.-L.C., M.L.E., S.S.K., J.C.S.M., E.C.G.-M., M.M., A.K.P., L.P., D.M.R., B.-J.S., R.V. and N.C.W. extracted descriptive information about the studies from their abstract and/or PDF using Google Forms (Table S1). Specifically, we extracted: (i) the journal and year of publication, (ii) the taxonomic group(s) studied, (iii) the climatic stressor(s) manipulated during the experiment(s), (iv) the life stage(s) exposed to the climatic stressor(s), (v) the life stage(s) of the animals when physiological or life-history traits were measured, (vi) the broad category of physiological or life history trait measured. Information on life stages were divided into three broad categories to allow cross-taxa comparisons: embryos, larvae or juveniles and adults. We did not separate larval and juvenile stages because we covered a broad range of taxa, and separations between these life stages were often unclear or difficult to establish based on study descriptions. For continuous experimental exposure overlapping multiple life stages, we noted if the climatic exposure was imposed on animals before and after hatching, or strictly after hatching. Climatic stressors were divided into five broad categories: (i) temperature, (ii) oxygen/carbon dioxide (O_2/_CO_2_), (iii) pH, (iv) salinity, (v) humidity/water availability and (vi) other (diet, UV radiation). We also noted if authors investigated interactions between climatic- and non-climatic stressors, although this was not one of our aims. Taxonomic groups were separated into seven broad categories: (i) birds, (ii) mammals, (iii) fish, (iv) non-avian reptiles, (v) amphibians, (vi) insects, (vii) other invertebrates. Trait categories were separated in seven broad classes: (i) environmental tolerance and preference (e.g. survival or tolerance to the climatic stressor, habitat selection, thermoregulation, heat shock proteins, etc.), (ii) energetics and metabolism (e.g. oxygen uptake, metabolic rate, aerobic scope, digestion efficiency, etc.), (iii) osmoregulation (e.g. ion balance, water loss, acid–base regulation, excretion, etc.), (iv) cardiovascular physiology (e.g. blood pressure, heart rate, stroke volume, etc.), (v) immune function and stress physiology (e.g. stress hormones, immune competence, oxidative stress, etc.), (vi) development (e.g. growth rate, body size, phenology, etc.) and (vii) reproduction (e.g. fecundity, sex hormones, gametogenesis, sperm count, etc.). We also collected whether studies investigated interactions between climatic and non-climatic stressors (e.g. predation). A few studies also reported data in trait categories we assigned as “Other” (e.g. thyroid function, neurophysiology, symbiont density, sensory physiology). Note that we also collected trait details within each category. The exact questions and response options presented in the Google form are presented in Table S1.

### Data curation visualization

Data were curated and visualized in R (R Core Team (2025)). PP curated the extracted data to amend typologies, and standardize trait, climatic stressor and life stage categories. All steps involved in the data curation are available at https://github.com/p-pottier/Cons_phys_life_stages. PP used the *tidyverse* collection of packages (Wickham *et al.*, 2019), *ggstream* (Sjoberg, 2025), *circlize* (Gu *et al.*, 2014) and *patchwork* (Pedersen, 2025) packages to produce the figures. We did not perform statistical analyses because our objective was to provide a systematic map of the current state of evidence.

#### Deviations from registration

While we mostly followed our original plans, we acknowledge one main deviation from our original plans: we did not perform bibliometric analyses to identify author clusters based on life stages. This was because the *bibliometrix* package (Derviş, 2019) does not currently allow for mapping of author clusters based on external variables, which prevented us from performing the planned analyses. Nevertheless, this analysis was not critical to our conclusions.

## Results

### Data description

**Figure 1:**
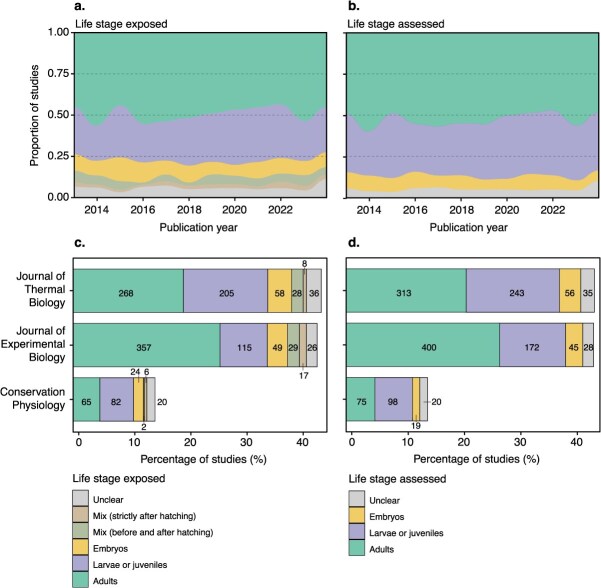
Differences in the relative percentage of life stages exposed to climatic stressor(s) (**a**) or assessed for physiological traits (**b**) over study publication years, and across the three representative journals surveyed (**c** exposed, and **d** assessed). Sample sizes (counts of studies) are presented for each category. Note that the values do not correspond to strict percentages of studies because some studies have investigated multiple life stages

We found 1276 studies measuring physiological and/or life-history responses of animals to climate change. All studies were published between 2013 and 2024 to maintain a consistent timespan across studies (Fig. 1a, b). Approximately 45% (*n* = 562) of studies were published in the *Journal of Experimental Biology*, 43% (*n* = 533) in the *Journal of Thermal Biology* and 15% (*n* = 181) in *Conservation Physiology* (Fig. 1c, d). Note that the values below do not represent a strict percentage of studies, because some studies have investigated multiple life stages, taxa, climatic stressors and traits. For clarity, we refer to percentages of studies rather than study entries.

**Figure 2:**
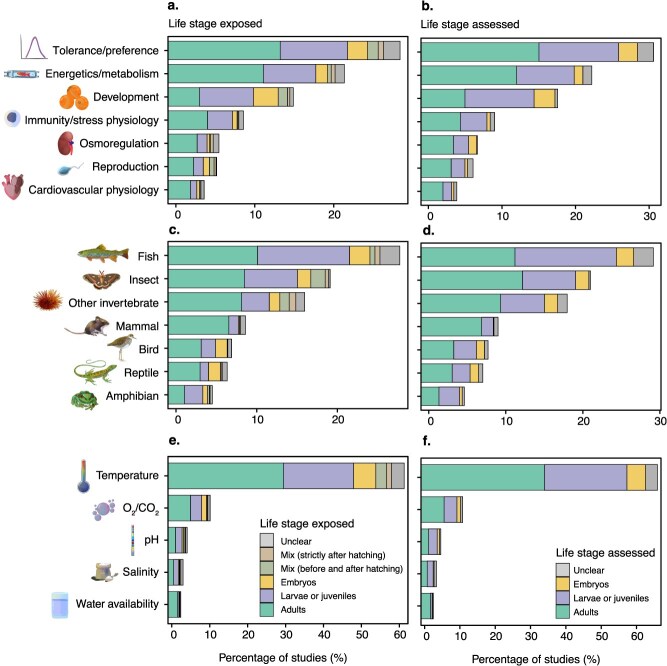
Differences in the relative percentage of life stages exposed to climatic stressor(s) (**a, c, e**) or assessed for physiological traits (**b, d, f**) across the traits (top row), taxa (middle row), and climate change stressors surveyed (bottom row). Studies categorized in “development” yet assessing adult animals are studies measuring whole animal size, growth or measuring multiple life stages. Other stressors not captured by these broad categories (i.e. diet, UV radiation; *n* = 6) and studies investigating interactions between climatic and non-climatic stressors (*n* = 138) are not displayed for clarity. Note that the values do not correspond to strict percentages of studies because some studies have investigated multiple life stages, traits, taxa or stressors. Representative diagrams were drawn by M.L.E.

We found that the eligible studies predominantly investigated environmental tolerance and preference (30.8%; *n* = 727) followed by energetics and metabolism (24.3%; *n* = 574), development (16.1%; *n* = 380), immune function and stress physiology (10.4%; *n* = 246), osmoregulation (7.2%; *n* = 170), reproduction (5.8%; *n* = 138) and cardiovascular physiology (5.2%; *n* = 122) (Fig. 2a, b). An additional 6 studies (0.3%) also investigated other traits not captured by these broad categories (e.g. sensory physiology, neurophysiology).

Studies covered a broad range of taxa, although we note that most of the data originated from fishes (31.1%; *n* = 398), insects (18.6%; *n* = 238) and other invertebrates (17.6%; *n* = 226). Mammals (10.7%; *n* = 137), birds (8.7%; *n* = 112), reptiles (7.8%; *n* = 100) and amphibians (5.5%; *n* = 70) were the least studied taxa in our literature sample (Fig. 2c, d).

Most studies investigated responses to temperature (65.1%; *n* = 1041), followed by O_2_/CO_2_ (12.1%; *n* = 194), pH (5.2%; *n* = 83), salinity (4.3%; *n* = 68) and humidity/water availability (3.8%; *n* = 60; Fig. 2e, f). Approximately 1% (*n* = 16) of studies investigated other stressors (i.e. diet, UV radiation) and 8.7% (*n* = 138) studied interactions between climatic and non-climatic stressors. The over-representation of studies on responses to temperature is, perhaps, not surprising because 45% of studies were published in the *Journal of Thermal Biology* (Fig. 1a, b). Nevertheless, temperature remained the most studied climate change stressor (54% of studies) after excluding studies published in this journal. Our results were also robust to the exclusion of environmental stressors other than temperature (Figs S2–S4).

### Variation in research effort across life stages

We found a strong bias towards the study of adults, larvae and juveniles relative to embryos. Specifically, only 9.4% (*n* = 131) of studies exposed embryos to climatic stressors, while 49.5% (*n* = 690) and 28.8% (*n* = 402) of studies exposed adults, or larvae/juveniles, respectively (Figs 1a, c and 2a, c, e). We also found that a notable percentage of studies exposed animals across different life stages, with 4.2% (*n* = 59) of studies exposing embryos and later life stages and 2.2% (*n* = 31) of studies exposing animals across different life stages strictly after hatching (Figs 1a, c and 2a, c, e). We also found that ~5.9% (*n* = 82) of studies did not clearly report which life stage was exposed to the climatic stressor (Figs 1a, c and 2a, c, e).

**Figure 3:**
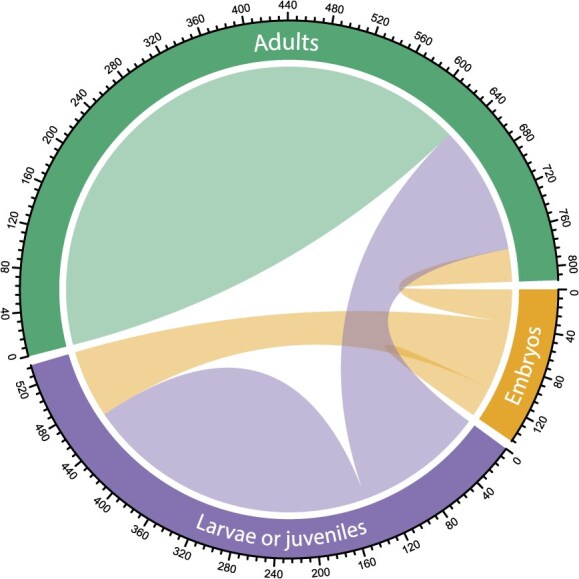
Chord diagram illustrating studies measuring traits on single or multiple life stages. Categories that are connected represent studies that investigated multiple life stages. Numbers in the outer circle represent the number of studies

We observed similar patterns for the life stages assessed for life-history or physiological traits (Figs 1b, d, 2b, d, f and 3). Most studies (52.4%; *n* = 788) measured the traits of adult animals, followed by larvae/juveniles (34.1%; *n* = 513) and embryos (8.0%; *n* = 120) (Fig. 3). About 5.5% (*n* = 83) also did not clearly report which life stage was assessed. Notably, we found that most studies reported the responses of single life stages, and few studies investigated the responses of multiple life stages (Fig. 3). Of studies that measured traits on adults (*n* = 788), only 18% (*n* = 142) and 4.6% (*n* = 36) also measured traits on larvae/juveniles and embryos, respectively (Fig. 3). Furthermore, 14.6% (*n* = 75) of studies measuring traits on larvae or juveniles (*n* = 513) also performed measurements on embryos (Fig. 3). Among the studies that specifically exposed embryos to climatic stressors (*n* = 84), 40.9% (*n* = 47) assessed potential carry-over effects in larvae or juveniles, while only 11.3% (*n* = 13) did so in adults.

Interestingly, biases towards the study of post-embryonic stages were consistent across time, and we did not observe a notable temporal change in research effort among life stages (Fig. 1a, b).

We also found consistent under-representation of studies on embryos across journals (Fig. 1c, d), taxonomic groups (Fig. 2c, d) and climate change stressors (Fig. 2e, f), for both the life stages exposed to the climatic stressors (Figs 1a, c and 2a, c, e) and those measured for physiological or life-history traits (Figs 1b, d and 2b, d, f). Patterns were also consistent among trait categories, with the exception of development, for which we found a larger proportion of studies on embryos, as expected (Fig. 2a, b). It is also worth noting that there were a few notable differences in research effort among taxonomic groups, with very few studies measuring traits on mammal embryos (*n* = 1), but significantly more in reptiles (*n* = 16; 13.7%; Fig. 2c, d).

## Discussion

Our systematic map revealed a pronounced and consistent bias in conservation physiology experimental research towards adult life stages, with embryos being largely underrepresented (8–9% of studies; Figs 1–3). This pattern was remarkably consistent across journals, taxa, physiological traits and climatic stressors, and has persisted over the past decade (Figs 1 and 2). The underrepresentation of embryos is especially concerning given the increasing recognition of their ecological importance and sensitivity to environmental stressors (Dahlke *et al.*, 2020; Wu and Seebacher, 2020; Sales *et al.*, 2021; Sun *et al.*, 2021; Vorsatz *et al.*, 2022; Pottier *et al.*, 2022b). Neglecting this vulnerable life stage risks obscuring key demographic bottlenecks and may lead to inaccurate predictions of species’ climate sensitivity, ultimately undermining the design of effective conservation strategies.

### Ontogenetic biases in conservation physiology research: historical, methodological and logistical constraints

Adult life stages were studied 1.5 to 1.7 times more than larvae and juveniles, and 5.3 to 6.6 times more than embryos in our literature sample (Figs 1–3). Studies that focused on embryos also remained significantly underrepresented across journals, taxonomic groups, physiological traits, climatic stressors and time, only representing between 1% and 15% of studies across all the axes we considered. The broad consistency of these patterns suggests that this study bias is not a product of editorial priorities or specific research areas, but instead reflects deep-rooted, structural limitations within scientific priorities and/or experimental design. Notably, this imbalance has persisted over the past decade, with no clear increase in embryo-focused studies, despite growing recognition in the ecological and developmental literature that early life stages often represent critical periods of heightened sensitivity to environmental change (Dahlke *et al.*, 2020; Wu and Seebacher, 2020; Sun *et al.*, 2021; Vorsatz *et al.*, 2022; Pottier *et al.*, 2022b; Vasudeva, 2023). The consistent ontogenetic bias in conservation physiology research likely reflects a combination of ‘historical’, ‘methodological’ and ‘logistical’ constraints that have shaped the field over time.

#### Historical bias

Ecological and conservation research has traditionally centered on juvenile and adult life stages, reflecting a prevailing view of fitness and population viability that emphasized survival, fecundity and performance in post-embryonic stages (e.g. Scott, 1994; Pechenik *et al.*, 1998; Lindström, 1999). Consequently, early physiological and ecological studies focused mainly on traits such as metabolic rate, stress tolerance, growth and reproductive performance in more developed life stages (e.g. McCay *et al.*, 1930; Gunn, 1942; Scholander *et al.*, 1950; Angilletta, 2009). This emphasis did not necessarily arise from explicit arguments about embryonic irrelevance; rather, it reflected the conceptual frameworks and methodological limitations of the time, during which the importance of early (i.e. embryonic) developmental stages was largely absent from discussions of population dynamics (Pechenik *et al.*, 1998). Conservation priorities were likely similarly influenced by this research focus, where population viability analyses and management strategies typically focused on juvenile and adult abundance, fecundity and survival rates (Mills *et al.*, 1999; Salguero-Gómez *et al.*, 2016). While these metrics remain critically important, such emphasis has inadvertently marginalized physiological research on early developmental stages, despite their large influence on recruitment, lifetime fitness and population persistence, as well as their potential to shape post-embryonic phenotypes and population trajectories through developmental plasticity and carry-over effects (Burggren and Mueller, 2015; Fawcett and Frankenhuis, 2015; Pettersen *et al.*, 2016; Noble *et al.*, 2018; Pottier *et al.*, 2022b). In turn, the underappreciation of the importance of early life stages in the study of environmental stressors has likely constrained access to research funding, further reinforcing ontogenetic biases in the published literature. Only more recently, has a life stage-inclusive perspective, recognizing that all life stages contribute to fitness in organisms with complex life cycles, become more widely articulated and integrated into ecological and conservation physiology research (e.g. Biek *et al.*, 2002; Benard and McCauley, 2008; Touchon *et al.*, 2013; Silla and Byrne, 2024).

#### Methodological constraints

Beyond historical biases, ‘methodological’ challenges have likely strongly influenced the underrepresentation of embryos in conservation physiology research. Embryonic life stages often present practical difficulties due to their small size, fragility and sensitivity to handling conditions, which complicate experimental design and physiological measurements, especially in small ectotherms. Many physiological traits require invasive or delicate techniques that are easier to implement in larger, more robust adults. For example, physiological assessments such as quantifying metabolic rates, hormone levels or thermal tolerance in embryos demand specialized equipment or techniques which can be logistically challenging or have only recently become more accessible (Ellis-Hutchings and Carney, 2010; Pettersen *et al.*, 2016; Truebano *et al.*, 2018; Wu *et al.*, 2020; Cowan *et al.*, 2023). Moreover, certain assessments such as stress hormone measurements from blood or tissue samples require a minimum sample mass or volume that embryos often do not meet (Burraco *et al.*, 2015; Ruthsatz *et al.*, 2023). Additionally, some embryos develop within protective structures such as egg capsules or maternal tissues, further limiting direct manipulation and observation. The need for species-specific breeding knowledge and controlled developmental environments may present further methodological obstacles. Successful breeding protocols and rearing techniques must often be established or optimized for each species, requiring substantial time and resources, especially for rare or threatened species. Moreover, many embryos have narrow developmental windows during which environmental manipulations can be applied without causing mortality or developmental arrest (Limpus *et al.*, 1979; Du and Shine, 2015), necessitating precise timing and monitoring. These constraints increase experimental complexity and costs which may discourage researchers from prioritizing embryonic stages despite their ecological importance. Recent advances in non-invasive imaging (Ibbini *et al.*, 2022; McCoy *et al.*, 2023; Tills *et al.*, 2025), molecular techniques (El-Ghali *et al.*, 2010; Wu *et al.*, 2020) and miniaturized instrumentation (Lovegrove, 2009; Cowan *et al.*, 2023) are beginning to address these challenges, offering new opportunities to study early-life physiology more effectively. Continued development and dissemination of standardized protocols will be essential to overcome methodological barriers and improve representation of embryos in conservation physiology research.

#### Logistical constraints

In addition to methodological constraints, ‘logistical’ factors may further limit the study of embryos from wild animals. Many early life stages occur in cryptic, transient or inaccessible habitats, complicating their detection, collection and experimental use. For instance, some aquatic species spawn in ephemeral pools or submerged substrates, while some terrestrial embryos may develop underground or within complex microhabitats, making field sampling labor-intensive and time-sensitive. Some taxa, such as anguillid eels, illustrate extreme logistical challenges where natural spawning grounds and embryonic stages remain poorly understood or unobservable (Tsukamoto, 2009), impeding ecologically relevant laboratory studies. Seasonality also constrains access to embryos, as reproductive events may be brief and highly synchronized (Lowerre-Barbieri *et al.*, 2011; Low, 2014), requiring precise timing for sample collection. These temporal limitations reduce flexibility in experimental design and can limit replication or longitudinal studies across developmental stages. Moreover, the need to maintain controlled environmental conditions during embryonic development adds logistical complexity, particularly for species with specific habitat requirements or sensitive life stages. In some species, thermal, moisture or chemical conditions required by early life stages to thrive can be hard to replicate in the laboratory, requiring equipment and techniques that may differ from those routinely used for adult life stages (El-Ghali *et al.*, 2010; Ellis-Hutchings and Carney, 2010). Collectively, these logistical challenges compound methodological difficulties, contributing to the pervasive underrepresentation of embryos in conservation physiology research. Addressing these barriers will require coordinated efforts to improve field sampling techniques, develop flexible rearing protocols and expand access to specialized laboratory equipment.

### Future recommendations and concluding remarks

Our findings highlight the need for a more life-stage inclusive approach in conservation physiology research. The strong and consistent underrepresentation of embryonic stages across journals, taxa, traits and climatic stressors suggests that this ontogenetic bias is pervasive and persistent, rooted in a combination of historical precedent and enduring methodological and logistical barriers. Addressing this gap is critical to improving the predictive power and ecological realism of conservation physiology research.

First, we recommend a deliberate shift towards understanding sensitivity to ecological stressors across multiple life stages, particularly embryos and larvae. Multi-stage studies can reveal hidden vulnerabilities that may be missed when focusing solely on adult stages. Several case studies illustrate how early developmental stages can fundamentally alter predictions of species persistence. For instance, in two invasive ascidians, fertilization and larval development proved far more sensitive to temperature, salinity and copper stress than later stages, such that successful development was not possible under conditions tolerated by adults, implying that population persistence depends on recruitment from elsewhere or brief benign windows (Pineda *et al.*, 2012). In a full life-cycle study of a glacial-relict butterfly, warming improved survival of multiple stages, yet negative effects on a single cryptic stage (overwintering larvae) drove all population-viability forecasts towards faster extinction under climate-change scenarios (Radchuk *et al.*, 2013). Similarly, demographic models for the threatened bog turtle show that improved egg and juvenile survival can buffer declines even in long-lived species where adult survival has the strongest elasticity (Knoerr *et al.*, 2022). Meta-analytic evidence also suggests that marine larvae tend to be more vulnerable to thermal stress and acidification than embryos, although effects varied largely among taxa and when multiple stressors were combined (Przeslawski *et al.*, 2015). Such examples demonstrate that early life stages can represent demographic bottlenecks or leverage points, and that their omission from research agendas can lead to inaccurate predictions of population responses to environmental change. Early-life exposures to thermal, nutritional or chemical stressors can also induce physiological carry-over effects that influence later-life performance, survival and reproductive success (Grandjean *et al.*, 2015; Noble *et al.*, 2018; Macartney *et al.*, 2019; Bodensteiner *et al.*, 2021; Wu *et al.*, 2025). However, among the few studies that specifically exposed embryos to climatic stressors, only 11% assessed potential carry-over effects in adulthood. Moreover, only 5% of studies on adults and 15% of studies on juveniles also measured traits on embryos (Fig. 3). Including underrepresented stages in physiological experiments would allow researchers to detect developmental trade-offs, identify critical windows of sensitivity and better pinpoint demographic bottlenecks. Conservation physiology would benefit from increased integration of such longitudinal and life-stage inclusive designs, where trait expression and fitness are understood as dynamic, cumulative processes shaped across the life cycle (Fawcett and Frankenhuis, 2015; Hodgson *et al.*, 2016; Marshall *et al.*, 2016). These perspectives are key, particularly when aiming to forecast population responses to environmental change or design targeted conservation measures.

Second, improving the feasibility of embryo-focused studies will require coordinated investment in tools, protocols and collaborations. Advances in non-invasive imaging (Keller *et al.*, 2008; Ibbini *et al.*, 2022;McCoy *et al.*, 2023 ; Tills *et al.*, 2025), molecular techniques (El-Ghali *et al.*, 2010; Wu *et al.*, 2020) and specialized instrumentation (Lovegrove, 2009; Kong *et al.*, 2016; Cowan *et al.*, 2023) have already begun to lower the technical barriers to studying small and fragile life stages. For example, non-invasive imaging techniques now allow for the acquisition of data undetectable to the naked eye. Eulerian video magnification can reveal imperceptible motion that can, for instance, be used to determine heart rate or embryo movement (Lauridsen *et al*., 2019; Ibbini *et al.*, 2022). In addition, analysis of pixels through automated recording systems now allows quantification of whole-organism embryonic phenotype (i.e. phenome), which can provide high-resolution indicators of physiological performance under different environmental stressors (McCoy *et al.*, 2023; Tills *et al.*, 2025). Recognition of the unique biology of embryos has also called for the standardization of methods across ontogeny (Pottier *et al.*, 2022a), giving birth to interesting new experimental techniques, for example, to quantify the thermal tolerance of fish embryos under ramping protocols (Cowan *et al.*, 2023). To accelerate progress, the field should prioritize the development and open sharing of standardized, scalable protocols tailored to embryonic and larval physiology, such as those developed in biomedical sciences and toxicology (Embry *et al.*, 2010; Busquet *et al.*, 2014). Promoting embryo-based research has already gained substantial traction in these fields, often serving as a foundational step preceding investigations of later developmental stages (Embry *et al.*, 2010; Busquet *et al.*, 2014). In fact, the Organization for Economic Co-operation and Development (OECD, 2013) has already established standardized guidelines and protocols specifically tailored for embryo studies, ensuring consistency and reproducibility across research efforts. These guidelines have significantly increased interest towards embryos in biomedical research since 2011 (Cassar *et al.*, 2019). Adapting similar guidelines to the context of conservation physiology would be an effective way for research efforts on embryos to gain traction.

Third, we encourage the consideration of embryo-focused studies to align with ethical considerations for animal experimentation, particularly the ‘Replace, Reduce, Refine’ (3Rs). In many jurisdictions, experimentation on vertebrate embryos is exempt from ethical permit requirements until specific developmental milestones—often the onset of independent feeding (e.g. fishes) or a defined stage of embryonic development (e.g. reptiles, birds; experiments on viviparous species, however, typically require additional ethical consideration). This framework reflects the principle that early embryos lack the neural development required for experiencing pain and therefore offer a meaningful way to apply the 3Rs. Embryonic stages allow researchers to explore fundamental physiological and ecological questions while reducing the use of sentient, fully developed individuals. Therefore, we see studies on embryos not only as an opportunity to tackle exciting research questions, but also as means to reduce reliance on sentient animals in experiments.

Fourth, while our synthesis focused on a subset of journals and physiological traits, the striking consistency of ontogenetic biases suggests our results may be generalizable to different contexts and journals. Nevertheless, future work should expand this mapping effort to a broader set of journals. Ontogenetic variation in research effort may vary between journals, particularly if some receive more or fewer embryo-focused submissions based on their thematic scope. However, in most journals, there is little reason to expect systematic editorial constraints that would limit the publication of studies based on life stages. Moreover, the consistent ontogenetic bias we observed across three distinct journals (Fig. 1c, d; Fig. S5) suggests this pattern is unlikely to be an artefact of journal choice, though it is worthwhile investigating. Navigating the conservation physiology literature across journals may prove difficult. Nonetheless, text-mining algorithms may help exploring ontogenetic patterns with narrower-focused questions than the present study (e.g. single taxon, trait and/or climatic stressor). We also recommend expanding future mapping efforts to other trait categories (e.g. behaviour, morphology), environmental stressors (e.g. toxicants), habitats (e.g. aquatic vs. terrestrial), life stage definitions (e.g. young larva vs. fully developed juvenile), as well as to include investigations of carry-over and transgenerational effects. Fine-scale resolution of these patterns would help clarify which taxa or subfields have generated the most knowledge, and where the greatest opportunities remain for growth. We also encourage more detailed reporting of life stages in experimental studies. We found that over 5% of studies did not specify the exact life stage used (Figs 1 and 2). While distinguishing life stages can be challenging in some species—especially when morphological changes are subtle and when ontogenetic variation is not a main study objective—providing clear descriptions of the likely life stage(s) used would improve interpretability and facilitate comparisons across studies.

Fifth, developing theoretical models for why differences in life stage-specific responses exist presents an extremely exciting avenue for research. Differences in life-stage specific response have been linked to size (Klockmann *et al.*, 2017; Medina-Báez *et al.*, 2023), mobility (e.g. pupal vs. mobile adults, Moghadam *et al.*, 2019), energy trade-offs between growth, maturation and reproduction (Makarieva *et al.*, 2004; Pörtner *et al.*, 2006; Sousa *et al.*, 2010) and the degree of phenotypic plasticity in response to a stressor (Walasek *et al.*, 2022; Pottier *et al.*, 2022b). Potential mechanisms have been proposed for differences in environmental sensitivity but not compressively assessed across the tree of life. For example, underdeveloped cellular repair mechanisms in developing insects (Bowler and Terblanche, 2008), tadpoles adjusting their energy investment under different stressors (Ruthsatz *et al.*, 2019) or mismatches between oxygen supply and demand in fishes (Dahlke *et al.*, 2020) are potential mechanisms. Across life stages, environmental cues also vary widely in their reliability and how they contribute to fitness, directly affecting the benefits and costs of changing phenotypes (Fawcett and Frankenhuis, 2015). A mixture of these mechanisms likely explains the variety of life-stage specific responses to ecological stressors, but elucidating shared responses among species can help conservation physiologists build better predictive models of species vulnerability to climate change.

In conclusion, the pervasive underrepresentation of early life stages in conservation physiology research likely reflects a legacy of structural constraints and adult-centric thinking that continues to shape research agendas. Yet, the biological rationale for studying embryos is compelling: they represent both a vulnerable life stage and a powerful window into the mechanisms of developmental plasticity, environmental sensitivity and long-term fitness. Failing to address these biases risks overlooking key demographic bottlenecks, ultimately limiting our ability to predict species' responses to global change and design effective conservation strategies. More ontogenetically inclusive research in conservation physiology is not only increasingly feasible thanks to emerging methods for measuring environmental sensitivity across life stages, but also essential for meeting the challenges of biodiversity loss and climate adaptation in the decades to come.

## Supplementary Material

Web_Material_coag006

## Data Availability

All data and materials used in this study are available publicly (https://github.com/p-pottier/Cons_phys_life_stages) and permanently archived in Zenodo (https://doi.org/10.5281/zenodo.18233973, Pottier *et al.*, 2026).
