# Supplementary material for: Embryos are largely understudied in a representative sample of journals in conservation physiology
Source: Conserv Physiol. 2026 Feb 18;14(1):coag006. doi: 10.1093/conphys/coag006 (PMC12916238; doi:10.1093/conphys/coag006)
Supplement: Web_Material_coag006 [file web_material_coag006.zip › Pottier_et_al_Con_Phys_supplementary_materials_revision_2.pdf]

Supplementary Information

TABLE OF CONTENTS

Figure S1 2
Figure S2 3
Figure S3 4
Figure S4 5
Figure S5 6
Table S1 7
Table S2 8
Table S3 9

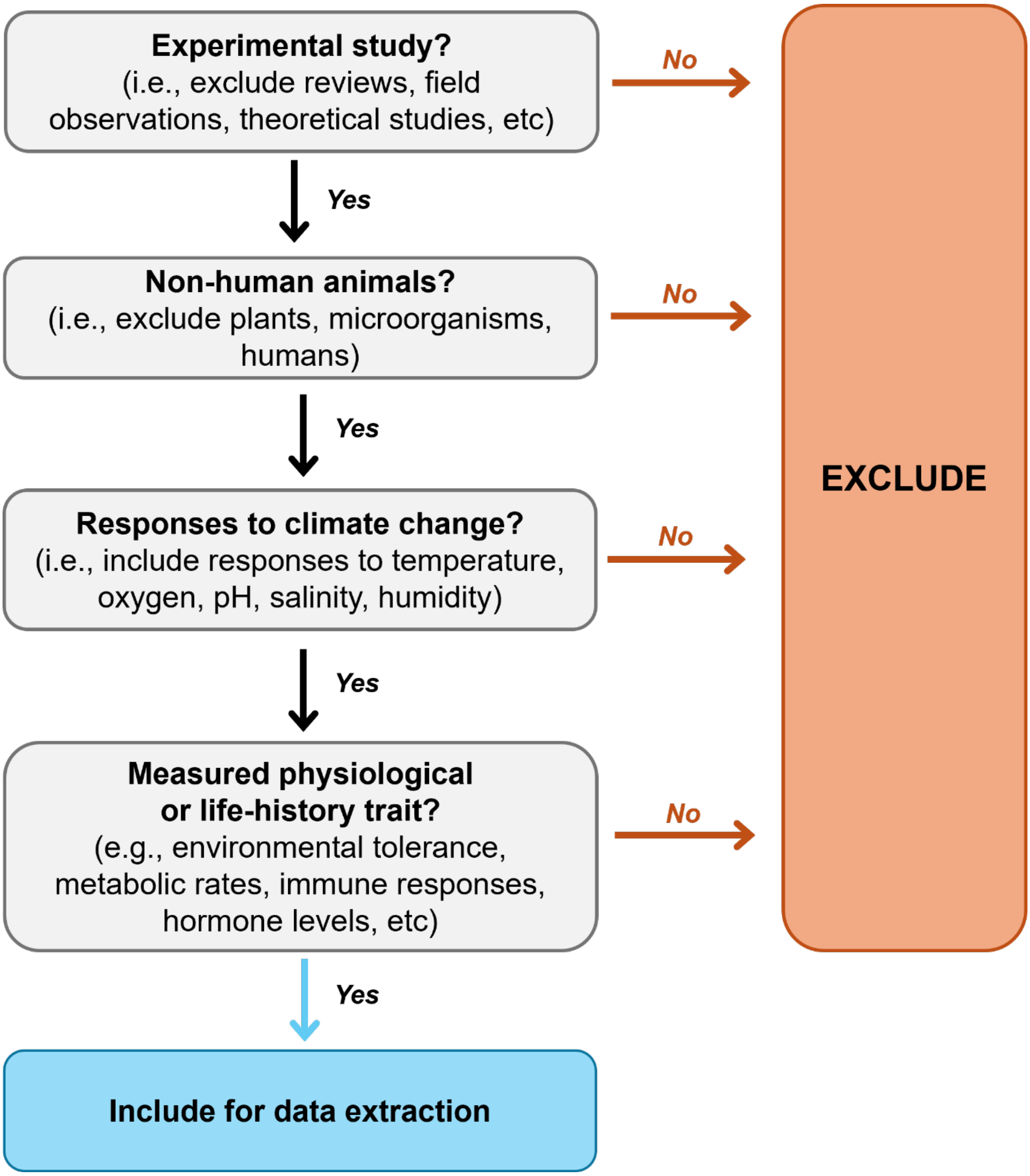

Figure S1: Decision tree used to screen studies for eligibility. When the title, abstract, or keywords provided insufficient detail to assess eligibility, the full article was examined.

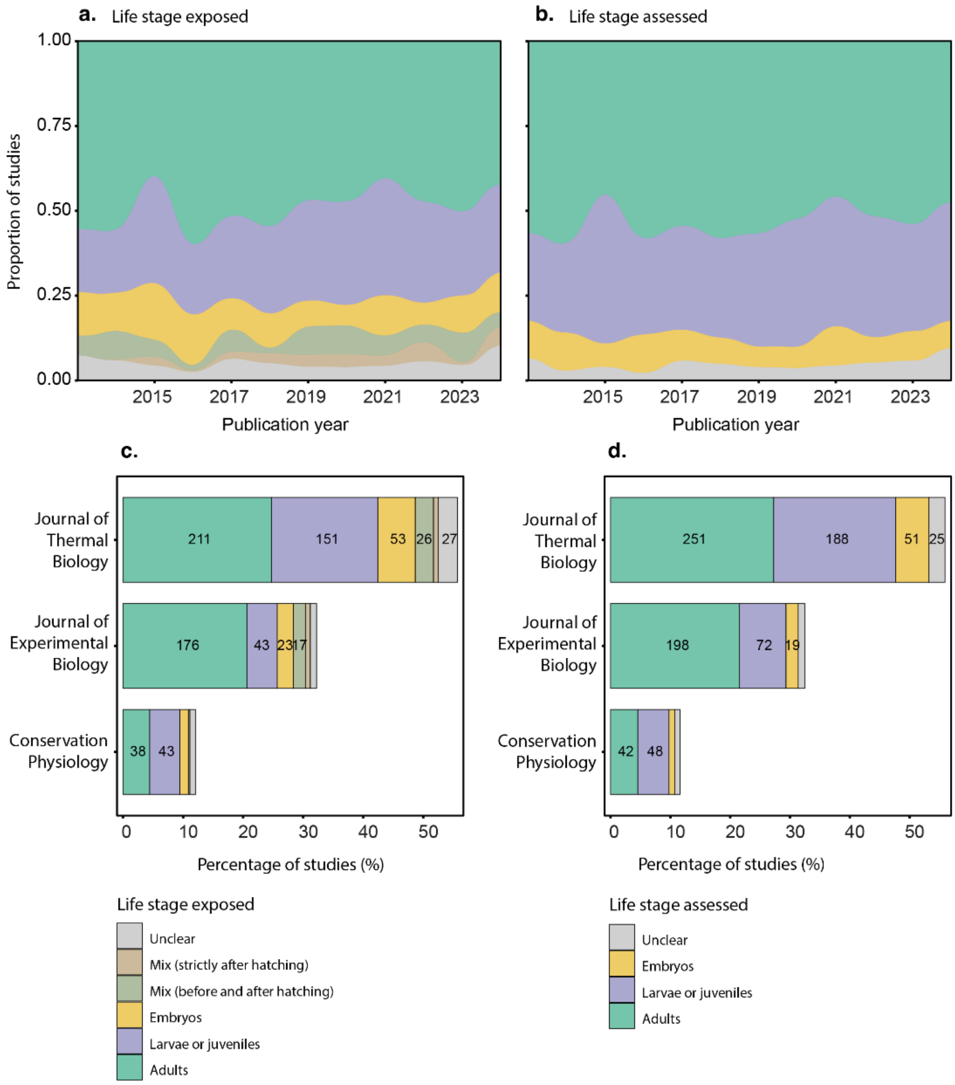

Figure S2: Differences in the relative proportion of life stages exposed to temperatures (**a**) or assessed for physiological traits after temperature exposures (**b**) over time, and across three representative journals surveyed (**c** exposed, and **d** assessed). Sample sizes (counts of studies) are presented for each category.

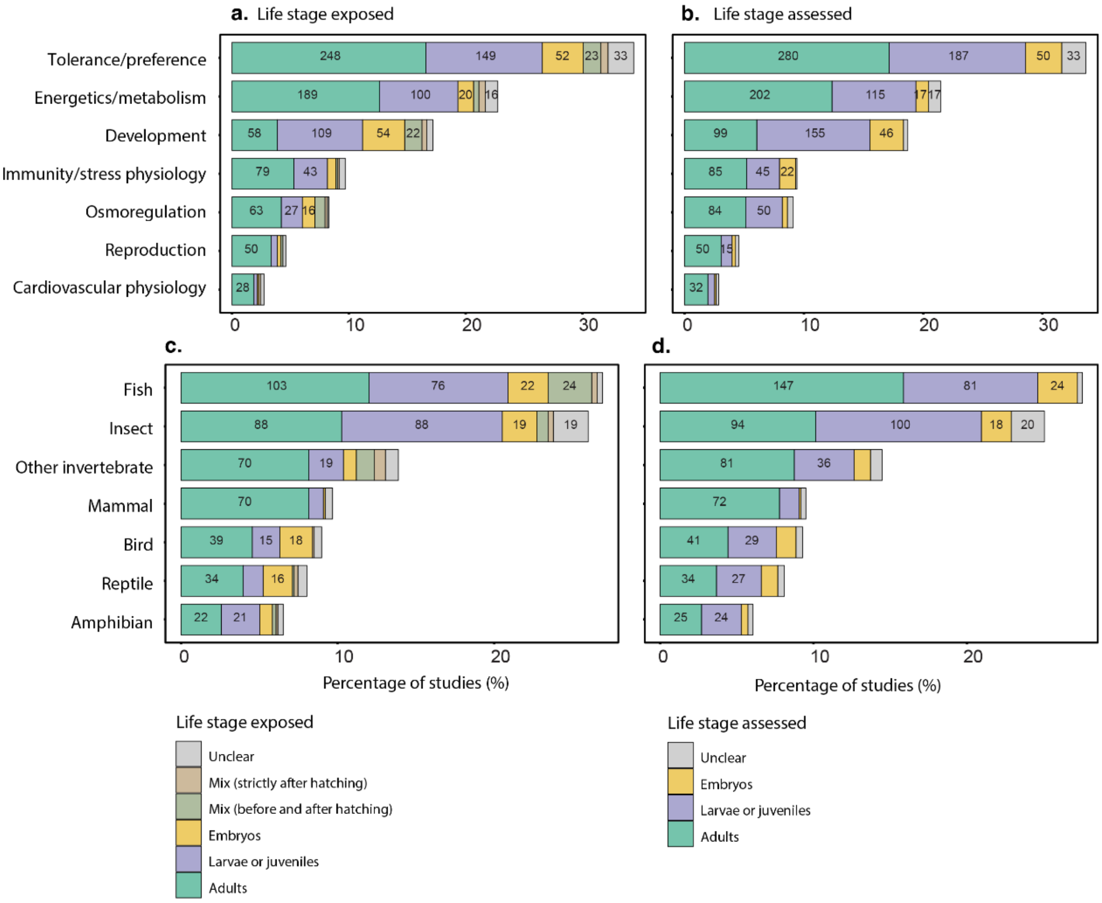

Figure S3: Differences in the relative proportion of life stages exposed to temperatures (**a, c, e**) or assessed for physiological traits after temperature exposures (**b, d, f**) across the traits (top row) and taxa (bottom row) surveyed.

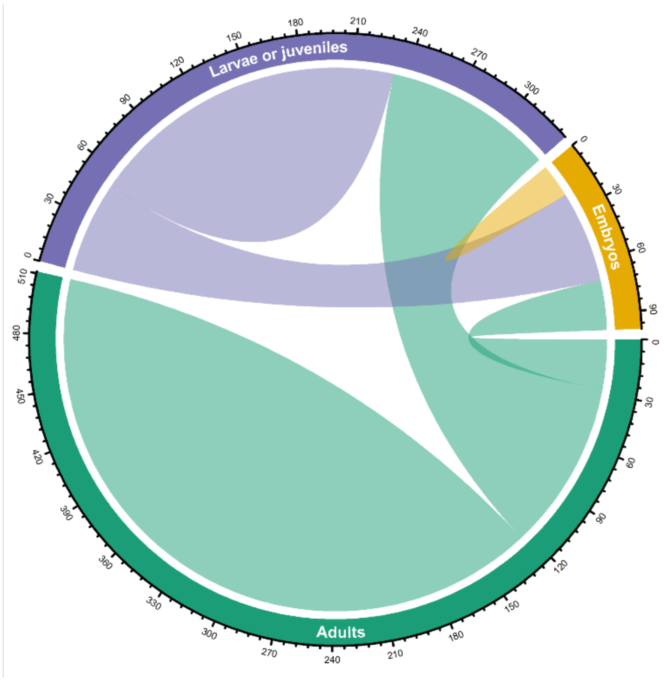

Figure S4: Chord diagram illustrating studies measuring traits on single or multiple life stages in a subset of studies on temperatures. Categories that are connected represent studies that investigated multiple life stages. Numbers in the outer circle represent the number of studies.

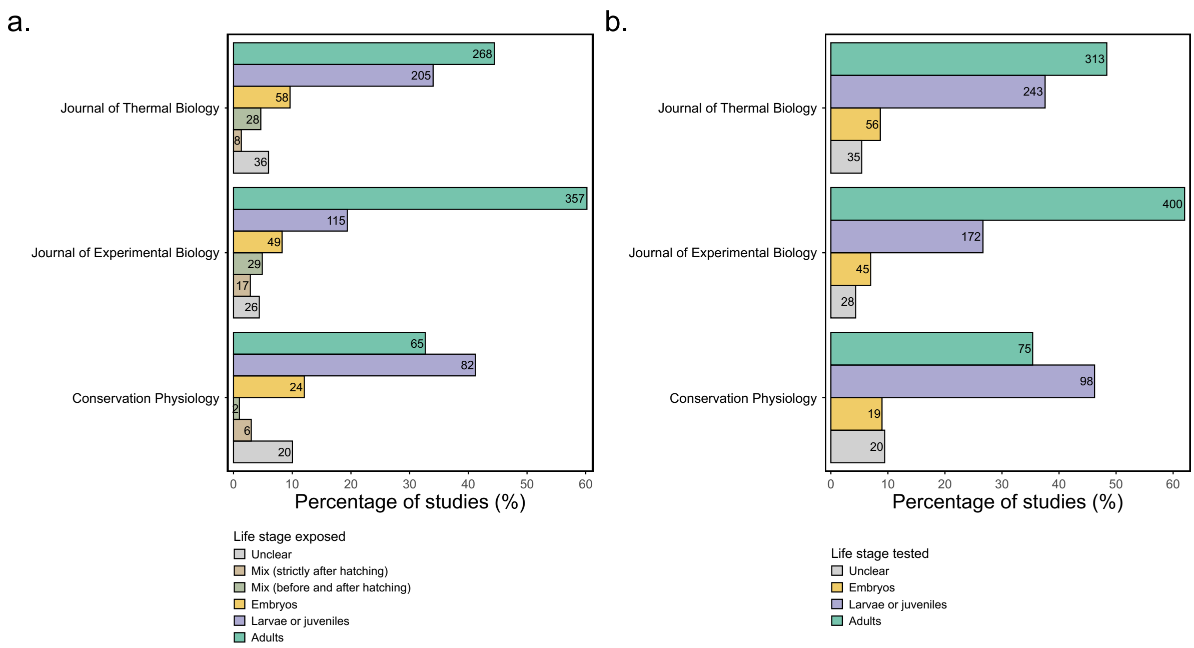

Figure S5: Variation in percentage of life stages exposed to temperatures (**a**) or assessed for physiological traits after temperature exposures (**b**) within each of the three journals surveyed. Sample sizes (counts of studies) are presented for each category.

Table S1: Questions and instructions used in Google Forms to facilitate data extraction. This form was distributed to all the researchers responsible for extracting data from studies.

Question	Description	Response options
Your initials	E.g., PP	Short-answer text
Short reference	Use FirstAuthorName_et_al_YEAR **for studies with 3 or more authors** (e.g., Ruthsatz_et_al_2024)<br>Use FirstAuthorName_and_SecondAuthorName_YEAR **for studies with 2 authors** (e.g., Ruthsatz_and_Pottier_2024)<br>Use FirstAuthorName_YEAR **for studies with a single author** (e.g., Ruthsatz_2024)	Short-answer text
Title	Copy and paste title directly from Rayyan - **do not type**.	Short-answer text
DOI	Copy and paste DOI directly from Rayyan - **do not type**.<br><br>4 studies do not have their DOI displayed in Rayyan. If you encounter one of these, please google the article and find the DOI. Paste the DOI without the URL (e.g., "10.1242/jeb.138784")	Short-answer text
Journal	NA	Conservation Physiology; Journal of Thermal Biology; Journal of Experimental Biology
Taxonomic group	If the authors have used multiple taxonomic groups (e.g., one predatory fish and one invertebrate prey), tick multiple boxes.	Bird; Mammal; Fish; Reptile; Amphibian; Insect; Other invertebrate.<br><br>Multiple responses allowed.
Climate change stressor	Tick the **climate change** stressor(s) the authors have **manipulated** during the experiments. Most studies manipulate a single stressor, but some may have used factorial designs with multiple stressors (e.g., temperature and acidification).<br><br>Please note that you can use the "Other" category for additional climatic stressors that are not captured below, but these must be direct climate change stressors (e.g., not pesticides, light pollution, urbanisation, etc).<br><br>If the authors used an experiment with an interaction between a climatic stressor **and** a non-climatic stressor (e.g., pollutant, disease, urbanisation), you can select "Interaction with non-climatic stressor". If they only used a non-climatic stressor, this study does **not** match our inclusion criteria.	Temperature; Oxygen; pH; Salinity; Humidity; Interaction with non-climatic stressor; Other (open text).<br><br>Multiple responses allowed.
Life stage exposed to the stressor	Here, select the life stage **exposed** to the manipulated stressor (temperature, oxygen, humidity, pH, or salinity).<br><br>If the authors performed separate experimental exposures on different life stages, select each life stage that applies. **However**, if the exposure **overlaps multiple life stages** (exposure from fertilisation to adulthood), select one of the "Mix" categories:	Embryos; Larvae or juveniles; Adults; Mix (before and after hatching); Mix (strictly after hatching); Unclear; Other (open text).<br><br>Multiple responses allowed.

	• "*Before and after hatching*" refers to exposures that started before hatching (e.g., exposure from eggs to adults),<br>• "*Strictly after hatching*" refers to exposures that started at the larval or juvenile stage.<br>It is often easy to tell based on the abstract (e.g., look out for words such as "adults", "larvae", or "juveniles). However, there will be cases when you will need to dive into the PDF.<br><br>Note also that there is a option "Unclear". Only use this option when the authors do not report what life stages were used, and these cannot be inferred.<br><br>Please do not include cases where cells or organs were isolated from the animals before the exposure to the climatic stressor. We are interested in the responses of whole living organisms to climatic stressors.	
Life stage of the animals when traits were measured	Here, select the life stage of the animals **when the traits of interest were measured**.<br><br>This can be different from the previous question. For instance, one study may incubate eggs at different temperatures and measure the oxygen consumption of embryos (in which case "embryos" should be selected in both questions). However, other studies may measure traits at a later point (e.g., at the juvenile stage) after incubating eggs to the climate change stressor (e.g., temperature).<br><br>If authors measure traits in multiple life stages, select all that apply.<br><br>Note also that there is a option "Unclear". Only use this option when the authors do not report what life stages were used, and these cannot be inferred.	Embryos; Larvae or juveniles; Adults; Unclear; Other (open text)<br><br>Multiple responses allowed
Trait category	**Environmental tolerance and preference** --> survival or tolerance to different stressors (temperature, pH, hypoxia, salinity), habitat selection, thermoregulation, heat shock proteins, etc.<br><br>**Energetics and metabolism** --> oxygen uptake, metabolic rate, aerobic scope, digestion efficiency, etc.<br><br>**Osmoregulation** --> Ion balance, water loss, acid-base regulation, excretion, etc.<br><br>**Cardiovascular physiology** --> Blood pressure, heart rate, stroke volume, etc.<br><br>**Immune function and stress physiology** --> stress hormones, immune competence, oxidative stress, etc.<br><br>**Reproduction** --> fecundity, sex hormones, gametogenesis, sperm count, etc.<br><br>**Development** --> growth rate, body size, phenology, etc.	Environmental tolerance and preference; Energetics and metabolism; Osmoregulation; Cardiovascular physiology; Immune function and stress physiology; Reproduction; Development; Other (open text).<br><br>Multiple responses allowed

	Use "Other" if none of these fit. **However**, remember that we are **only** interested in physiological and life-history traits, so most relevant traits are likely to fit into these categories. If authors measured physiological and/or life-history traits along with other traits (e.g., behaviour, morphology), do not use the "Other" category to add additional traits that are not relevant to our study.<br><br>For instance, we are **not** interested in behavioural traits (e.g., dispersal, exploration, activity, learning, cognition, etc), morphological traits (e.g., body shape, abnormalities, pigmentation, coloration, etc), ecological interactions (e.g., predator-prey interactions, symbiosis, microbiome diversity), biodiversity parameters (e.g., abundance, species richness, heterozygosity), etc.<br><br>Although we don't include morphological traits, note that we include measures of whole animal size (e.g., body size, body mass, snout-vent length, size at metamorphosis etc) in the "Development" category.<br><br>Although we excluded cases where cells or organs were isolated from the animals before the exposure to the climatic stressor, we include physiological traits measured on cells/organs taken from living organisms during/after the exposure to the climatic stressor.	
Trait details	Indicate the specific traits that were measured (as described by the authors), **separated by semi colons.**<br><br>For example, "development time; oxygen consumption".	Long-answer text
Additional comments	If you have important comments, please indicate them here. Otherwise, leave this question blank.	Long-answer text

Table S2: Authorship contributions according to the Dragon Kill Points guidelines (Martinig et al. 2025).

**Contribution**	**Author initials**
Conference session organisation	PP, NCW, KR
Communication with the journal Conservation Physiology	PP, KR
Study conceptualisation	PP, KR
Study design feedback	PP, ML, KR
Pre-registration (original draft)	PP
Pre-registration (review & editing)	PP, ML, KR
Literature searches	PP
Literature screening	NCW, MLE, ML, KA, AC, ZLC, SSK, JCSM, ECGM, RA, MM, LP, AKP, DMR, BS, RV
Data extraction	NCW, MLE, ML, KA, AC, ZLC, SSK, JCSM, ECGM, RA, MM, LP, AKP, DMR, BS, RV
Data extraction quality checks	PP, KR
Data cleaning and processing	PP
Figures (original draft)	PP
Figures (feedback)	NCW, MLE, KR
Figures (cosmetic adjustments)	PP, NCW, MLE
Manuscript introduction and discussion (original draft)	KR
Manuscript methods and results (original draft)	PP
Manuscript introduction and discussion (second draft)	PP, NCW, KR
Manuscript methods and results (second draft)	PP, NCW, KR
Manuscript (review and editing)	PP, NCW, MLE, ML, KA, AC, ZLC, SSK, JCSM, ECGM, RA, MM, LP, AKP, DMR, BS, RV, KR
Project administration	PP, KR

Table S3: Authorship contribution points, according to the Dragon Kill Points guidelines (Martinig et al. 2025)

**Author initials**	**Authorship points ("Dragon Kill Points")**
PP	16
NCW	8
MLE	5
ML	5
KA	3
AC	3
ZLC	3
SSK	3
JCSM	3
ECGM	3
RA	3
MM	3
LP	3
AKP	3
DMR	3
BS	3
RV	3
KR	12
